# Epidemiology of asphyxiation suicides in the United States, 2005–2014

**DOI:** 10.1186/s40621-017-0131-x

**Published:** 2018-01-08

**Authors:** Rebecca K. Yau, Mallie J. Paschall

**Affiliations:** 1 Prevention Research Center, Pacific Institute for Research and Evaluation, 180 Grand Avenue, Suite 1200, Oakland, CA 94612 USA; 2 School of Public Health, University of California, Berkeley, 50 University Avenue, Berkeley, CA 94720 USA

**Keywords:** Suicide, Epidemiology, Asphyxiation

## Abstract

**Background:**

From 2005 to 2014, the asphyxiation suicide rate in the United States (U.S.) increased by 45.7% from 2.45 to 3.57 per 100,000 population. The primary purpose of this cross-sectional study was to describe decedent and incident characteristics of asphyxiation suicides in the U.S. from 2005 to 2014. The secondary purpose of this study was to explore whether any demographic characteristics of asphyxia suicide decedents were associated with type of suicide incident.

**Methods:**

Data from the National Violent Death Reporting System (NVDRS) were used to describe asphyxiation suicide mechanisms and means in 16 states. Anchor points of hanging suicides were also described. Mechanisms, means, and anchor points were determined through a text search of cause of death, coroner/medical examiner narrative, and law enforcement narrative. Multivariable logistic regression was conducted separately for females and males to estimate beta coefficients to obtain adjusted odds ratios (AORs) and 95% confidence intervals (CIs) to compare hanging-related asphyxiation and other types of asphyxiation.

**Results:**

From 2005 to 2014, there were 25,270 asphyxiation suicides. Most decedents were male (79.9%) and white, non-Hispanic (76.8%). Most asphyxiation suicides involved hanging (90.7%, *N* = 22,931); 1717 (6.8%) involved smothering; 968 (3.8%) involved chemicals or gasses; and 145 (0.6%) involved strangulation. For hanging suicides, the three most commonly used means were power or extension cords (*N* = 1834), bedding (*N* = 873), and animal ropes (*N* = 578). The three most common anchor points for hanging suicides were trees (*N* = 2215), beams (*N* = 2014), and closets (*N* = 2009). Among females and males, odds of asphyxiation suicide were highest among those of Other, non-Hispanic race and black, non-Hispanic race, respectively [AOR (95% CI) = 3.73 (1.59, 8.79) and 2.72 (1.34, 5.50), respectively].

**Conclusions:**

Commonly available objects are used in asphyxiation suicides. Modification of anchor points represents a potential solution for reducing hanging suicides. Changes in design and availability of grocery bags could help reduce smothering suicides. Strategies to reduce asphyxiation suicides need to be identified. Improving access to and utilization of mental health services can also reduce asphyxiation suicides. Future research should be conducted to better describe characteristics of asphyxiation suicide so that prevention efforts targeted by demographic subgroups can be implemented.

## Background

In 2014, suicides were the tenth leading cause of death in the United States (U.S.). Among the 42,773 suicides in 2014, 11,407 (26.7%) were due to hanging, strangulation, or suffocation (collectively referred to as asphyxia for the remainder of this paper), the second leading cause of suicides behind firearms (Centers for Disease Control and Prevention). From 2005 to 2014, the asphyxia suicide rate increased by 45.7% from 2.45 to 3.57 per 100,000 population, while the absolute number of asphyxia suicides increased by 57.4% (Centers for Disease Control and Prevention). In 2010, asphyxiation suicides in the U.S. cost the economy $13.1 billion in medical and work loss costs, or $1.38 million per suicide (Centers for Disease Control and Prevention). These suicides also leave an economic and emotional burden on surviving family members and friends (Cerel et al. 2008, Jordan and McIntosh 2011).

Asphyxiation suicides can be categorized into three types: suffocation, neck compression, and involvement of chemicals or gasses. During suffocation, the oxygen supply to the body is restricted by limiting the air supply in the environment (Dix et al. 2000; Prahlow 2010). Two subtypes of suffocation are 1) smothering, where the air supply is restricted by covering the nose or mouth and 2) choking, where the internal airway is blocked by foreign objects (Dix et al. 2000, Prahlow 2010). Neck compression occurs when a force is placed on the neck that compresses prominent blood vessels or airways and consists of two subtypes: 1) hanging, where a ligature places a force through the victim’s body weight and 2) strangulation, where a force other than body weight is exerted on the neck (Dix et al. 2000, Prahlow 2010). In asphyxiation involving chemicals or gasses, the chemical or gas prevents the oxygen from either being transported in the blood or being used by the cells (Dix et al. 2000, Prahlow 2010).

Previous research has investigated overall (Nock et al. 2008) and firearm (Santaella-Tenorio et al. 2016) suicides extensively, but limited research has been conducted on asphyxia suicides. One previous study in the U.S. described the epidemiology of asphyxiation suicides, and noted that asphyxiation suicide rates increased from 2000 to 2010; the increase varied by age (Baker et al. 2013). Furthermore, in 2010, asphyxiation suicide rate peaked at ages 25–44 at 4.6 per 100,000 population, with decreased asphyxiation suicide rates at both younger and older ages (Baker et al. 2013). One previous systematic review specifically investigated the epidemiology of hanging suicides (i.e., not all asphyxiation suicides), and concluded that reductions in suicide hangings could be achieved by focusing on reducing hanging suicides in institution settings (e.g., prisons, hospitals) and reducing the lethality of attempted suicides through enhanced emergency management procedures (Gunnell et al. 2005). However, no prior studies have examined the distribution of asphyxia suicide means and mechanisms, and how these are related to decedent demographic characteristics. Doing so will improve understanding of how and where asphyxiation suicides occur in the U.S., and population subgroups at risk for specific types of asphyxia suicide. Describing such characteristics may help to identify strategies to prevent asphyxia suicides. Therefore, the primary purpose of this study was to describe distribution and characteristics of asphyxia suicide decedents and incidents. The secondary purpose of this study was to explore whether any demographic characteristics of asphyxia suicide decedents were associated with type of suicide incident. Based on previous findings, we expect asphyxiation suicide rates to be highest among those ages 25–44 years (Baker et al. 2013), males (Callanan and Davis 2011), and white, non-Hispanic decedents (Callanan and Davis 2011).

## Methods

The current cross-sectional study used 2005–2014 National Violent Death Reporting System (NVDRS) data for 16 states; NVDRS characteristics have previously been described (Crosby et al. 2016, Paulozzi et al. 2004). Briefly, NVDRS is an active surveillance system that gathers information on all homicides, suicides, legal intervention deaths, and firearm-related deaths. Its primary data sources are coroner/medical examiner (CME) records, crime laboratory records, law enforcement (LE) records, and death certificates (Crosby et al. 2016, Paulozzi et al. 2004). At its inception in 2002, six states were NVDRS participants (Paulozzi et al. 2004). As of 2016, 40 states, the District of Columbia, and Puerto Rico were NVDRS participants (Centers for Disease Control and Prevention).

In the present study, through searching for key terms in CME narratives, LE narratives, and causes of death, we categorized asphyxiation into the following types: smothering, swallowing a foreign object (i.e., choking), hanging, strangulation, and chemical. A suicide incident could result from multiple types of asphyxiation.

Solely for the purposes of investigating potential differences in distribution of asphyxiation type by demographic characteristics, we investigated asphyxiation suicides resulting only from one specific type of asphyxiation (as opposed to multiple types of asphyxiation). Furthermore, we limited our investigation to asphyxiation suicides resulting from smothering, hanging, strangulation, or chemicals. The total number of decedents from other types of suicide was low (*N* = 19).

Potential asphyxiation suicides were identified by selecting incidents where manner of death was suicide and the primary method used to inflict the injury was coded “hanging, strangulation, or suffocation”. Per NVDRS coding rules, deaths due to smoke inhalation were excluded, as these should have been coded “fire or burn”. Additionally, any deaths due to carbon monoxide (CO) were excluded, as these deaths should have been coded “poisoning”. We also excluded any deaths where CO was not explicitly mentioned, but the decedent was in an automobile in an enclosed space and the car ignition was turned on when the decedent was found. To identify potential miscodes, one author (RKY) reviewed CME and LE narratives for records where the cause of death mentioned car exhaust, CO, or fire. Additionally, CME and LE narratives for records where the cause of death mentioned drowning or firearms were reviewed. There were sixty-five suicides that were identified where the primary method was not “hanging, strangulation, or suffocation” (35 where either CO poisoning was involved or the victim died in a car and it was specifically mentioned that the car ignition was on, 14 where the victim died in a fire, eight due to gunshot wounds, seven due to drowning; and one due to other poisoning).

We identified asphyxiation means (e.g., rope, plastic bag) through record review conducted by one author (RKY) on a subset of records. First, 200 records were randomly selected for review to identify the means. After all means mentioned were identified, we reviewed additional records in increments of 100 to identify additional means. Once we reached a group of 100 asphyxiations where no new means were identified, we ended record review. In total, 2400 records were reviewed to identify asphyxiation means. Additionally, to code for potential chemicals used in chemical asphyxiation, we searched for terms identified by Azrael et al. (Azrael et al. 2016) and also noted any additional chemicals found in our review of 2400 records. Concurrently with identifying asphyxiation means, we identified anchor points of hanging suicides (e.g., beam, light fixture). During the review process, we also identified one additional type of asphyxiation suicide: through removal of medical care.

After record review was complete, we developed an algorithm that searched for key terms identified in the record review to code for asphyxiation means and locations of hanging suicides. To perform a quality check on algorithm coding, one author (MJP) reviewed 200 randomly selected records to code for asphyxiation mechanism and means. Additionally, hanging suicide records were reviewed for anchor points. The overall agreement was high (166/200, 83.0%, Cohen’s Kappa = 0.65). Of the 34 records with discrepancies between the reviewer and algorithm, ten were related to hanging anchor points, ten were related to hanging means, and six were related to identification of smothering as a mechanism or means of smothering. The remaining eight discrepancies were related to misspellings (e.g., the word “electRIc” was spelled “electIRc” in the narrative) or punctuation marks not incorporated in the algorithm. Location of asphyxiation suicide was determined using an existing NVDRS data field with 33 possible locations (including “Other” and “Unknown”). We combined and reduced to 15 locations to minimize the number of categories with less than five suicides.

### Statistical analysis

To calculate asphyxiation suicide rates, U.S. bridged-race population estimates produced by the U.S. Census Bureau and National Center for Health Statistics were obtained from the Centers for Disease Control and Prevention (CDC) Wide-ranging Online Data for Epidemiologic Research (Centers for Disease Control and Prevention). We used negative binomial regression (to account for overdispersion) to calculate model-based 10-year incident rate ratios (IRRs) and 95% confidence intervals (CIs) to compare the incident rates of asphyxiation suicides between 2005 and 2014. To investigate potential differences in distribution of asphyxiation type by demographic characteristics, Pearson’s chi-square tests were conducted for bivariate analysis to investigate associations with each characteristic individually.

Additionally, multivariable logistic regression was conducted to estimate beta coefficients to obtain adjusted odds ratios (AORs) and 95% CIs to compare hanging-related asphyxiation to other types of asphyxiations. Based on previous findings that suicide characteristics differ by gender (though these findings were not specific to asphyxiation suicides) (Callanan and Davis 2011), multivariable modeling was conducted separately for females and males. We also considered models both with and without education status, since nearly half (44.8%) of decedents were missing education status. Additionally, to increase the power to detect potential effects in our multivariable models, we combined age categories to reduce the number of categories from nine categories to five categories. Finally, we used ages 21–34 years as our as the referent category as this category had the greatest number of suicides in our five-category age variable, and therefore would produce the smallest standard errors that would allow for more precise 95% CIs in the AORs. Results were considered statistically significant at *p* < 0.05.

Data analyses were performed in SAS 9.4. Results with counts fewer than five were suppressed, per CDC guidelines.

## Results

### Overall

From 2005 to 2014, there were 25,270 asphyxiation suicides in 16 NVDRS states. Most decedents were male (79.9%) and white, non-Hispanic (76.8%) (Table 1). The overall 10-year asphyxiation-related suicide IRR (95% CI) was 1.47 (1.39, 1.57) (Table 1) This increase varied by demographic subgroup. Most asphyxiation suicides involved hanging (90.7%, *N* = 22,931), 1717 (6.8%) involved smothering, 968 (3.8%) involved chemicals or gasses, 145 (0.6%) involved strangulation, 14 (<0.1%) involved swallowing a foreign object, and less than five involved removing medical care. 2.0% of asphyxiation suicides (*N* = 508) involved none of these specific types of asphyxiation, 94.0% (*N* = 23,757) involved one type of asphyxiation, 3.7% (*N* = 939) involved two types of asphyxiation, and 0.3% (*N* = 66) involved three types of asphyxiation. There were 291 (1.2%) asphyxiation-related suicides where no additional information in either the narrative portions of the CME or LE reports were available for determining specific means related to the suicide.

**Table 1:** Demographic characteristics of asphyxiation suicide decedents, United States 2005–2014

	Total	Column %	2005		2006		2007		2008		2009		2010		2011		2012		2013		2014		10-year IRR (95% CI)
			N	Rate	N	Rate	N	Rate	N	Rate	N	Rate	N	Rate	N	Rate	N	Rate	N	Rate	N	Rate	
Total	25,270	100.0%	1966	2.9	2045	3.0	2256	3.3	2292	3.3	2545	3.6	2647	3.7	2716	3.8	2813	3.9	2815	3.8	3175	4.3	1.47 (1.39, 1.57)
Sex ^a^																							
Male	20,203	79.9%	1574	5.1	1681	5.4	1858	5.9	1877	5.9	2048	6.3	2110	6.5	2162	6.6	2208	6.6	2227	6.6	2458	7.2	1.40 (1.31, 1.48)
Female	5066	20.0%	392	1.1	364	1.0	398	1.1	414	1.1	497	1.3	537	1.4	554	1.4	605	1.5	588	1.5	717	1.8	1.85 (1.64, 2.08)
Race																							
White, NH	19,402	76.8%	1471	3.0	1555	3.2	1685	3.4	1772	3.6	1984	4.0	2021	4.1	2104	4.2	2202	4.4	2156	4.3	2452	4.9	1.62 (1.51, 1.74)
Black, NH	1732	6.9%	158	1.6	153	1.5	163	1.6	152	1.4	157	1.5	183	1.7	172	1.6	167	1.5	209	1.8	218	1.9	1.24 (1.05, 1.47)
Asian/PI, NH	667	2.6%	^c^	4.1	^c^	5.9	39	8.6	59	4.9	65	5.7	^c^	6.7	^c^	4.8	^c^	7.7	77	6.9	^c^	7.0	1.67 (1.27, 2.18)
Other, NH ^b^	1306	5.2%	97	^d^	124	^d^	152	^d^	127	^d^	136	^d^	150	^d^	146	^d^	137	^d^	121	2.3	^d^	^d^	^d^
Hispanic	2124	8.4%	188	3.3	165	2.7	212	3.4	174	2.6	203	3.0	223	3.1	222	3.0	220	2.9	247	3.2	270	3.4	1.08 (0.92, 1.26)
Unknown, NH	39	0.2%	^c^	^d^	^c^	^d^	5	^d^	8	^d^	0	^d^	^c^	^d^	^c^	^d^	^c^	^d^	5	^d^	^c^	^d^	^d^
Age (years)																							
10–14	494	2.0%	^c^	0.9	^c^	0.8	^c^	0.6	38	0.7	^c^	0.8	50	0.9	^c^	0.9	56	1.0	62	1.1	^c^	1.2	1.67 (1.23, 2.27)
15–19	2029	8.0%	178	3.2	166	2.9	173	3.0	198	3.4	206	3.5	215	3.7	200	3.5	228	4.0	217	3.9	248	4.4	1.49 (1.28, 1.74)
20–24	2873	11.4%	279	5.1	250	4.5	253	4.5	238	4.3	286	5.1	289	5.0	312	5.3	324	5.4	302	5.0	340	5.6	1.21 (1.06, 1.37)
25–29	2761	10.9%	212	4.1	235	4.5	268	5.0	241	4.4	274	4.9	301	5.4	291	5.1	319	5.6	312	5.5	308	5.3	1.33 (1.17, 1.52)
30–39	5083	20.1%	405	3.7	443	4.1	503	4.7	451	4.2	516	4.8	483	4.5	524	4.8	553	5.1	571	5.2	634	5.7	1.47 (1.33, 1.62)
40–49	5419	21.4%	441	3.7	474	3.9	538	4.5	526	4.4	556	4.7	589	5.0	606	5.2	584	5.0	519	4.5	586	5.2	1.38 (1.20, 1.58)
50–59	4084	16.2%	214	2.1	248	2.4	281	2.7	388	3.6	442	4.0	425	3.8	469	4.1	464	4.0	543	4.6	610	5.1	2.42 (1.97, 2.96)
60–69	1464	5.8%	78	1.3	95	1.5	108	1.6	122	1.7	126	1.7	170	2.2	156	1.9	188	2.2	172	2.0	249	2.7	1.96 (1.58, 2.43)
70+	1039	4.1%	103	1.6	90	1.3	96	1.4	90	1.3	95	1.4	114	1.6	107	1.5	97	1.3	117	1.5	130	1.6	1.10 (0.89, 1.35)
Unknown	24	0.1%	^c^	^d^	^c^	^d^	^c^	^d^	0	^d^	^c^	^d^	11	^d^	^c^	^d^	0	^d^	0	^d^	^c^	^d^	^d^
Education																							
Less than high school	3850	15.2%	312	^d^	361	^d^	346	^d^	306	^d^	403	^d^	429	^d^	388	^d^	357	^d^	379	^d^	569	^d^	^d^
High school graduate	5524	21.9%	332	^d^	381	^d^	425	^d^	484	^d^	515	^d^	610	^d^	622	^d^	673	^d^	600	^d^	882	^d^	^d^
Some college	2724	10.8%	153	^d^	172	^d^	199	^d^	205	^d^	218	^d^	286	^d^	334	^d^	343	^d^	287	^d^	527	^d^	^d^
College graduate	1906	7.5%	105	^d^	115	^d^	158	^d^	130	^d^	181	^d^	215	^d^	242	^d^	228	^d^	197	^d^	335	^d^	^d^
Unknown	11,898	47.1%	1148	^d^	1073	^d^	1181	^d^	1168	^d^	1247	^d^	1107	^d^	1131	^d^	1225	^d^	1501	^d^	1117	^d^	^d^

*Abbreviations: CI* confidence interval, *IRR* incident rate ratio, *NH* non-Hispanic, *PI* Pacific Islander

^a^There was one decedent of unknown sex

^b^Includes American Indian/Alaska Native, Other, and Two or more races

^c^Cell counts suppressed due to counts fewer than five deaths

^d^Rate and IRR not calculated because of lack of population estimates for demographic group

Among 23,757 asphyxiation suicides involving one type of asphyxiation, 22,663 involved hanging, 784 involved smothering, 168 involved chemicals or gasses, 134 involved strangulation, and 8 involved swallowing a foreign object. The distribution of asphyxiation type varied by demographic groups. Among females, 6.8% of asphyxiation suicides were due to smothering, where as 2.4% were related to smothering among males. Additionally, hanging accounted for a smaller proportion of suicides (94.7%) among white, non-Hispanic decedents, compared to those of other racial and ethnic backgrounds. As age increased, the proportion of asphyxiation suicides related to hanging decreased, while the proportion related to smothering and chemicals or gasses generally increased (Table 2).

**Table 2:** Demographic characteristics of asphyxiation suicide decedents by mechanism, United States 2005–2014

	Total		Hanging		Smothering		Chemical		Strangulation		χ2	*p*-value
	N	%	N	%	N	%	N	%	N	%		
Total	23,749	100.0%	22,663	95.4%	784	3.3%	168	0.7%	134	0.6%		
Sex (N unknown = 1)											223.6	<0.0001
Male	19,034	100.0%	18,316	96.2%	465	2.4%	138	0.7%	115	0.6%		
Female	4714	100.0%	4346	92.2%	319	6.8%	30	0.6%	19	0.4%		
Race (N unknown = 34)											125.4	<0.0001
White, NH	18,083	100.0%	17,116	94.7%	722	4.0%	141	0.8%	104	0.6%		
Black, NH	1676	100.0%	1639	97.8%	17	1.0%	^b^	^b^	^b^	^b^		
Hispanic	2065	100.0%	2031	98.4%	20	1.0%	^b^	^b^	^b^	^b^		
Other, NH ^a^	1891	100.0%	1845	97.6%	24	1.3%	11	0.6%	12	0.6%		
Age (years, N unknown = 15)											1423.0	<0.0001
10–14	483	100.0%	480	99.4%	^b^	^b^	0	0.0%	^b^	^b^		
15–19	1969	100.0%	1941	98.6%	^b^	^b^	5	0.3%	^b^	^b^		
20–24	2729	100.0%	2657	97.4%	48	1.8%	15	0.5%	9	0.3%		
25–29	2621	100.0%	2542	97.0%	44	1.7%	19	0.7%	16	0.6%		
30–39	4811	100.0%	4671	97.1%	86	1.8%	28	0.6%	26	0.5%		
40–49	5096	100.0%	4887	95.9%	139	2.7%	37	0.7%	33	0.6%		
50–59	3780	100.0%	3554	94.0%	157	4.2%	37	1.0%	32	0.8%		
60–69	1314	100.0%	1224	93.2%	66	5.0%	16	1.2%	8	0.6%		
70+	931	100.0%	693	74.4%	222	23.8%	11	1.2%	5	0.5%		
Education (N unknown = 10,630)											48.0	<0.0001
Less than high school	3698	100.0%	3638	98.4%	42	1.1%	6	0.2%	12	0.3%		
High school graduate	5273	100.0%	5106	96.8%	117	2.2%	14	0.3%	36	0.7%		
Some college	2504	100.0%	2358	94.2%	101	4.0%	19	0.8%	26	1.0%		
College graduate	1644	100.0%	1459	88.7%	144	8.8%	34	2.1%	7	0.4%		

*Abbreviations: NH* non-Hispanic

^a^Includes Asian/Pacific Islander, American Indian/Alaska Native, Other, and Two or more races

^b^Cell counts suppressed due to counts fewer than five deaths

Table 3 presents multivariable logistic regression results, stratified by gender. Since nearly half (44.8%) of decedents were missing information on education, we explored whether those missing information on education differed than those not missing education information. Decedents missing information on education were not different than those not missing education with respect to gender (χ^2^_1_ = 3.34, *p* = 0.07), but did differ with respect to age (χ^2^_4_ = 72.68, p = <0.0001), race (χ^2^_3_ = 43.65, *p* < 0.0001), and type of asphyxiation suicide (χ^2^_3_ = 15.59, *p* = 0.001; results not shown). However, since the point estimates for the AORs were approximately the same for both race and age regardless of whether or not we included education in the models, we present results only for the models including education. Among females, the odds of an asphyxiation suicide being hanging-related were highest among those of Other, non-Hispanic race [AOR (95% CI) = 3.73 (1.59, 8.79), reference = White, non-Hispanic race] and with less than high school education. Among males, the odds of asphyxiation suicide being hanging-related were highest among those of black, non-Hispanic race [AOR (95% CI) = 2.72 (1.34, 5.50), reference = White, non-Hispanic race] and with less than high school education.

**Table 3:** Odds of Hanging suicides compared with other asphyxiation suicides in the U.S., 2005–2014

	Male		Female	
	AOR (95% CI)	*p*-value	AOR (95% CI)	*p*-value
Race (ref = white, NH)				
Black, NH	2.72 (1.34, 5.55)	<0.01	0.85 (0.38, 1.91)	0.69
Hispanic	2.47 (1.37, 4.44)	<0.01	2.25 (0.87, 5.83)	0.09
Other, NH	1.82 (1.14, 2.89)	0.01	3.73 (1.59, 8.79)	<0.01
Age (ref = 21–34 years)				
10–20	1.20 (0.72, 1.98)	0.48	1.50 (0.58, 3.88)	0.40
35–49	0.91 (0.68, 1.21)	0.52	0.72 (0.42, 1.22)	0.22
50–64	0.95 (0.69, 1.31)	0.76	0.31 (0.19, 0.52)	<0.01
65 or older	0.26 (0.18, 0.36)	<0.01	0.04 (0.02, 0.07)	<0.01
Education (ref = less than high school degree)				
High school degree	0.64 (0.45, 0.92)	0.02	0.61 (0.33, 1.16)	0.13
Some college	0.30 (0.20, 0.43)	<0.01	0.39 (0.20, 0.74)	<0.01
College graduate	0.18 (0.12, 0.26)	<0.01	0.27 (0.14, 0.51)	<0.01

*Abbreviations: AOR* adjusted odds ratio, *CI* confidence interval, *NH* non-Hispanic, *ref.* reference

The five most common locations for asphyxiation suicides to occur were house or apartment (19,182, 75.9%); park, playground, natural area, or public use area (1447, 5.7%); jail, prison, or detention facility (1336; 5.3%), hotel or motel (679, 2.7%); and a hospital, medical facility, or supervised residential facility (410, 1.6%; Table 4). The location of asphyxiation suicides varied by mechanism of asphyxiation. Most notably, smothering and chemical suicides had increased proportions occurring in a hotel or motel; hanging suicides had increased proportions occurring in parks/public use areas and correctional facilities.

**Table 4:** Asphyxiation suicide location, United States 2005–2014

	Overall		Hanging		Smothering		Chemical	
	N	Column %	N	Column %	N	Column %	N	Column %
Total	25,270	100.0%	22,931	100.0%	1717	100.0%	968	100.0%
House or apartment	19,182	75.9%	17,540	76.5%	1316	76.6%	713	73.7%
Park, playground, natural area, or public use area	1447	5.7%	1404	6.1%	31	1.8%	14	1.4%
Correctional facility	1336	5.3%	1291	5.6%	16	0.9%	0	0.0%
Hotel or motel	679	2.7%	480	2.1%	162	9.4%	120	12.4%
Hospital, medical facility, or supervised residential facility	410	1.6%	348	1.5%	41	2.4%	5	0.5%
Street, sidewalk, or alley	249	1.0%	227	1.0%	18	1.0%	13	1.3%
Industrial area, construction area, or abandoned building	207	0.8%	199	0.9%	5	0.3%	6	0.6%
Commercial establishment	207	0.8%	201	0.9%	6	0.3%	5	0.5%
Farm	177	0.7%	165	0.7%	^a^	^a^	^a^	^a^
Child care center or school	142	0.6%	131	0.6%	11	0.6%	5	0.5%
Motor vehicle	134	0.5%	43	0.2%	57	3.3%	57	5.9%
Office building	70	0.3%	59	0.3%	7	0.4%	7	0.7%
Public parking lot or garage	68	0.3%	51	0.2%	13	0.8%	10	1.0%
Other	631	2.5%	598	2.6%	23	1.3%	9	0.9%
Unknown	331	1.3%	194	0.8%	^a^	^a^	^a^	^a^

^a^Cell counts suppressed due to counts fewer than five deaths

### Hanging

Among 22,931 hanging suicides, 11,045 (48.2%) did not mention any type of means, 6783 (30.0%) mentioned a general word for a ligature (e.g., belt, chain, rope, sheet, without further explanation as to the type of ligature used), and 5103 (22.3%) mentioned a specific means. There were 78 hanging suicides (0.3%) that involved chemicals or gasses. The most commonly used means were: power or extension cords (*n* = 1834), bedding (*n* = 873), animal ropes (e.g., lariat, dog leash; *n* = 578), clothing (*n* = 456), and belts (e.g., leather belt, karate belt, bathrobe belt; *n* = 315), shoelaces (*n* = 281), plastic bags (*n* = 156), towels (*n* = 121), clotheslines (*n* = 114), tow ropes (*n* = 65), telephone cords (*n* = 65), and bungee cords (*n* = 60).

We also investigated anchor points for hanging. The five most commonly mentioned anchor points were trees (*N* = 2215), beams (e.g., rafter, pipe, *N* = 2014), closets (2009), ceiling fans (*N* = 194), door knobs (*N* = 187), vents (*N* = 178), and door frames (*N* = 104).

### Smothering

Among 1717 smothering suicides, 159 were identified through mention of the word “smother” in the cause of death or narrative fields, and the remaining 1558 were identified by any mention of placing an object or over the head or face. There were 93 smothering suicides (5.4%) did not mention any type of means, 98 (5.7%) mentioned a general word for either a bag or sheet, without any further description of those objects. Nearly half (42.8%, *n* = 735) of smothering suicides involved chemicals or gasses. The most common objects used for smothering were: plastic bags (*n* = 1458), bedding (*n* = 59), and clothing (*n* = 27). The most commonly identified objects that could be used to secure the specific means of smothering were tape (*n* = 277) and zip ties (*n* = 21).

### Chemical

Nine hundred sixty eight (3.8%) asphyxiation suicides involved chemicals or gasses. Twenty-one of these suicides involved multiple chemical or gas. The most frequently mentioned chemicals or gasses were helium (*n* = 778), nitrogen (*n* = 49), bromine (*n* = 26), nitrous oxide (*n* = 26), propane (*n* = 26), carbon dioxide (*n* = 21), methane (*n* = 20), and hydrogen sulfide (*n* = 10), freon (*n* = 8), argon (*n* = 6), and difluoroethane (*n* = 5).

### Strangulation

One hundred fourty five (0.1%) of asphyxiation suicides involved strangulation. 58 (40.0%) did not mention any type of means, 23 (15.9%) mentioned a general word for a ligature. Less than five strangulation suicides involved chemicals or gasses. The most commonly used means were zip ties (*n* = 28), bedding (*n* = 9), plastic bags (*n* = 6), clothing (*n* = 6), and power or extension cords (*n* = 5).

## Discussion

In our study of 16 NVDRS states, from 2005 to 2014 we found that the asphyxiation-related suicide rate increased by 47% from 2.9 to 4.3 per 100,000. This increase differed across demographic subgroups and was highest among females; Asian/Pacific Islander, non-Hispanic individuals; and adults aged 50–69 years. One potential reason for the overall increases in asphyxiation-related suicide rates is the increasing popularity of internet use and social media during our study. For example, the social networking site Facebook had nearly 1 million users in 2004, and increased to 700 million users in 2011 (Luxton et al. 2012). Furthermore, a positive association has been previously found in overall suicide rates and prevalence of internet use (Luxton et al. 2012). Two possible ways that internet use and social media can increase risk of asphyxiation suicides include cyberbullying and greater access to information providing descriptions on how to commit suicide (Biddle et al. 2008, Luxton et al. 2012). However, the internet and social media can also be beneficial in preventing suicides through providing information on where to obtain support or counseling (e.g., the National Suicide Prevention Lifeline) (Biddle et al. 2008, Luxton et al. 2012, Robinson et al. 2016).

The majority of asphyxiation suicides were hangings that occurred among males and white, non-Hispanics; these demographic subgroups had the highest asphyxiation suicide rates. Although one prior study investigating asphyxiation suicides did not specifically calculate suicide rates, this study found that male and white decedents had the highest odds of asphyxiation suicide (Callanan and Davis 2011). One possible reason for the higher rates of asphyxiation suicides is because these groups of people may use methods and means of asphyxiation suicide that tend to be more lethal. A prior study investigating the case fatality rates (CFRs) of all suicides (i.e., not limited to asphyxiation suicides) found that males had a higher CFR than females (15.8% vs. 3.3%), and white, non-Hispanics had a higher CFR than other racial/ethnic groups (9.8%, vs. 5.6%–7.5% for all other groups) (Spicer and Miller 2000).

Similar to previous findings (Baker et al. 2013), those ages 20–49 had the highest asphyxiation suicide rates in the current study. These higher rates are likely unrelated to increased lethality of suicide mechanisms and means used among decedents in these age groups. If suicide rates were directly related to CFR, we would expect older adults to have the highest suicide rates, as prior research has demonstrated that CFR of suicide increases as age increases (Spicer and Miller 2000). Instead, these higher rates are likely due to a combination of: 1) high overall suicide rates among those ages 20–49 (ranging from 15.1–19.2 per 100,000 population in 2015 in the U.S., vs. overall suicide rates for all ages = 13.3 per 100,000; Centers for Disease Control and Prevention) and 2) a higher proportion of suicides being related to asphyxiation for those ages 20–49 (ranging from 28.8–37.1% in 2015 in the U.S., vs. 26.8% for all ages; Centers for Disease Control and Prevention).

In the current study, among both males and females, the odds of an asphyxiation suicide being hanging-related varied by demographics. Over three-quarters (75.9%) of asphyxiation suicides occurred in a house or apartment; 5.3% occurred in a correctional facility; and 1.6% occurred in a hospital, medical facility, or supervised residential facility. A variety of means were used in the asphyxiation suicide, and hanging suicides involved different anchor points.

Prior research has been conducted on strategies to reduce suicides overall (Van et al. 2011, Zalsman et al. 2016), and such methods would also be applicable to asphyxiation related suicides. However, most of the remaining discussion focuses on potential prevention efforts relevant specifically to asphyxiation suicides. One strategy generally used for suicide prevention is means restriction (Daigle 2005, Yip et al. 2012). However, this strategy is unlikely to succeed in preventing asphyxiation suicides. In the current study, over 90% of asphyxiation suicides were hanging-related. Among the hanging incidents where a means was identified, commonly available objects (e.g., power cords, bedding, clothing) were used in the hanging. Similarly, commonly available objects were used in smothering and strangulation suicides.

For hanging suicides, one potential solution for reducing these suicides would be focusing on modifying the anchor points from which people hang themselves on. Although many hanging suicides occur on an anchor point meant to support heavy weights (e.g., beams on a building, tree branch), some occur on anchor points not designed to support heavy weights for long periods of time (e.g., ceiling fan, vent). Therefore, it could be possible to modify the design of these items such that the items detach from where they are connected to after a heavy weight is placed on them for a protracted time period. However, products designed in such a manner would need to meet any laws, regulations, or standards established by agencies such as the Consumer Product Safety Commission.

Another avenue towards preventing a small proportion hanging suicides is to focus on institutional settings where environments can be controlled. A prior systematic review focused on hanging suicides, and discussed ways that hanging suicides could potentially be reduced in institutional settings. Suggestions included that clothing that do not need belts or shoes that do not have laces (e.g., using velcro straps) be issued to people in the institutions (Gunnell et al. 2005). In the current study, 6% (*N* = 1486) of all suicides occurred in a medical or correctional facility. Fifty-four of these suicides (3.6%) involved use of belts or shoelaces. Hanging suicides involving these objects could have been prevented if the apparel provided at these facilities did not have belts or shoelaces.

In the current study, 1458 out of 1717 smothering suicides (84.9%) involved plastic bags. One option for reducing smothering suicides is to modify the design of plastic bags by increasing the permeability of plastic bags such that it is easier for air to flow in and out of the bag. For example, plastic grocery bags are objects designed solely for transporting groceries more easily between locations, and increasing the permeability of the bag does not necessarily change the utility of the plastic grocery bags. However, increasing the permeability of bags designed for storage (e.g., plastic sandwich bags for food storage) is not a viable option, as these products are designed specifically with the intent of keeping air outside of the space of the bag. In the current study, we did not distinguish between different types of plastic bags used in the smothering suicide. Nonetheless, changing the design of plastic bags represents a strategy not previously explored for reducing smothering suicides. Another option related specifically to plastic grocery bags is to reduce the availability of these bags through policy interventions (e.g., taxes or bans on plastic grocery bags) intended to alter consumer behaviors (Ritch et al. 2009).

### Strengths and limitations

Our study had several strengths. First, we used multiple years of NVDRS data and over 25,000 asphyxiation suicides, minimizing the possibility that our results are due to random variation in small numbers. Additionally, to the best of our knowledge, our study was the first to describe suicide means for all asphyxiation suicides, and not only for hanging suicides. We also calculated suicide rates to ensure that the increase in asphyxiation suicide counts observed were not solely due to increases in population size.

Despite our study’s strengths, there were a few key limitations. First, our study results cannot necessarily be generalized to the entire U.S., as we only used data from 16 states that were part of NVDRS for all ten years of our study. In our study of 16 NVDRS states, we found that the asphyxiation-related rate increased from 2.9 per 100,000 in 2005 to 4.3 per 100,000 in 2014. Nationwide, this rate increased from 2.5 per 100,000 to 3.6 per 100,000 (Centers for Disease Control and Prevention). Currently, NVDRS is the only data source available for multiple states in the U.S. that incorporates death certificate data and rich information from CME and LE records, and therefore is the best data source for this current study.

Additionally, in identifying means and anchor points for asphyxiation suicides, we used an algorithm that identified key words or phrases for the specific means and anchor points. However, simply because a keyword was identified in a given record, it did not necessarily mean that the means (or anchor point) associated with that keyword was truly a means (or anchor point). For example, it was possible that there was a hanging suicide where a suicide note was placed in a plastic ziploc bag, and the bag would have been identified as the hanging means by our algorithm despite the fact it was not truly one of the means used. Nonetheless, we believe our study results are relatively robust, as we conducted a reliability check by manually reviewing 200 asphyxiation suicide records, and there was perfect agreement in 82.9% of the records between the algorithm and the reviewer. Refinements were made to the algorithm code to improve precision and reliability. With adequate resources, future studies could improve on our study by manually reviewing all asphyxiation suicides for means and anchor points, similar to a previous study conducted on gas suicides (Azrael et al. 2016).

## Conclusions

Strategies to reduce asphyxiation suicides need to be identified, especially given the increase in these incidents from 2005 to 2014 in the U.S and the high economic costs of suicides. Means restriction as an overall strategy for reducing these suicides would likely be met with limited success, given the variety of common objects used for these suicides. One potential avenue for reducing hanging suicides is to change the design of objects commonly used as anchor points, such that these objects collapse if a large weight is placed on them for an unusually long time period. Improvements in access to and utilization of mental health services for individuals at elevated risk for suicide is also needed to address this public health problem. Any intervention designed to reduce asphyxiation suicides should be tailored by demographic subgroups and by gender. Future research should conduct more extensive work around the epidemiology of asphyxiation suicides to better describe characteristics of these suicides so that targeted prevention strategies can be implemented.

## Abbreviations

AOR: Adjusted odds ratio
CDC: Centers for Disease Control and Prevention
CFR: Case fatality rate
CI: Confidence interval
CME: Coroner/medical examiner
LE: Law enforcement
NVDRS: National Violent Death Reporting System
U.S.: United States
